# No Specific Gene Expression Signature in Human Granulosa and Cumulus Cells for Prediction of Oocyte Fertilisation and Embryo Implantation

**DOI:** 10.1371/journal.pone.0115865

**Published:** 2015-03-13

**Authors:** Tanja Burnik Papler, Eda Vrtacnik Bokal, Luca Lovrecic, Andreja Natasa Kopitar, Ales Maver

**Affiliations:** 1 Department of Human Reproduction, Division of Gynaecology, University Medical Centre Ljubljana, Slajmerjeva 3, SI-1000 Ljubljana, Slovenia; 2 Clinical Institute of Medical Genetics, Division of Obstetrics and Gynaecology, University Medical Centre, Slajmerjeva 3, SI-1000 Ljubljana, Slovenia; 3 Faculty of Medicine, Institute of Microbiology and Immunology, University of Ljubljana, Zaloska 4, SI-1000 Ljubljana, Slovenia; Center for Human Reproduction, UNITED STATES

## Abstract

In human IVF procedures objective and reliable biomarkers of oocyte and embryo quality are needed in order to increase the use of single embryo transfer (SET) and thus prevent multiple pregnancies. During folliculogenesis there is an intense bi-directional communication between oocyte and follicular cells. For this reason gene expression profile of follicular cells could be an important indicator and biomarker of oocyte and embryo quality. The objective of this study was to identify gene expression signature(s) in human granulosa (GC) and cumulus (CC) cells predictive of successful embryo implantation and oocyte fertilization. Forty-one patients were included in the study and individual GC and CC samples were collected; oocytes were cultivated separately, allowing a correlation with IVF outcome and elective SET was performed. Gene expression analysis was performed using microarrays, followed by a quantitative real-time PCR validation. After statistical analysis of microarray data, there were no significantly differentially expressed genes (FDR<0,05) between non-fertilized and fertilized oocytes and non-implanted and implanted embryos in either of the cell type. Furthermore, the results of quantitative real-time PCR were in consent with microarray data as there were no significant differences in gene expression of genes selected for validation. In conclusion, we did not find biomarkers for prediction of oocyte fertilization and embryo implantation in IVF procedures in the present study.

## Introduction

*In vitro* fertilization (IVF) has been the main therapy for infertility for over 30 years however, despite great efforts its success remains limited [1]. To improve the chances for pregnancy in IVF procedures multiple embryos are being transferred and this results in a higher rate of multiple gestations and an increase in maternal and perinatal morbidity and mortality [2]. To prevent adverse outcomes related to multiple pregnancies elective single embryo transfer (SET) is increasingly being used in IVF procedures. Currently, the criteria for oocyte and embryo selection are morphological. In oocytes, the selection is based on the assessment of the polar body, meiotic spindle, zona pellucida and cytoplasm whereas embryo parameters include pronucleuar oocyte morphology, the time to the entry into the first mitotic division, fragmentation rate, blastomere number and morphology [3]. However, there is growing evidence, that the subjective morphological assessment alone does not accurately predict oocyte's developmental potential and embryos with the highest chance of implantation [4,5]. Thus, non-invasive, objective and reliable markers that could identify the most quality oocytes and embryos with the highest implantation potential and would not compromise the success of IVF procedures in SET are needed.

The presence of mature and quality oocyte in human IVF procedures is crucial not only for fertilization but also for subesquent embryo development [6]. Oocyte maturation and competence are acquired during follicular development where granulosa (GC) and cumulus (CC) cells play an essential role [7]. The oocyte plays a dominant role in regulating GC and CC functions during folliculogenesis [8, 9] and it is therefore believed that functions of GC and CC indirectly reflect oocyte's competence. Cell functions and active cell processes are regulated through gene expression therefore, gene expression analysis in GC and/or CC could provide a non-invasive method for identification of the most competent oocytes and embryos. These cells are easily accessible and discarded during IVF procedures and can be sampled without compromising the oocyte.

In recent years, several studies have tried to identify candidate genes expressed in somatic cells of ovarian follicles that could be used as biomarkers of oocyte and embryo quality [10,11] and successful embryo implantation [12,13,14,15]. However, the genes proposed as potential biomarkers show little overlap between different study groups. The reason for the discrepancy could be in the different endpoint or study design. Furthermore, it is known that factors such as the stimulation protocol and patient's characteristics influence gene expression [16,17]. Besides that, global gene expression profiling methods can give false positive results [18] and rigorous statistical analyses are needed in order to determine differentially expressed genes with greater accuracy [19,20].

Given this background, markers that would predict oocyte competence more accurately than morphological assessment alone, regardless of the patient, cycle and embryo culture characteristics, are still to be found.

In this study we aimed to determine potential gene expression signatures in GC and CC that could be used for the prediction of embryo implantation and oocyte fertilisation. For this purpose a prospective study of genome wide gene expression analysis was performed in GC and CC gained during classic IVF procedures.

## Materials and Methods

### Patient population and IVF procedure

Forty-one IVF patients were included in this study. The study was approved by the National Medical Ethics Committee of the Republic of Slovenia and all patients have signed informed consent prior to participation. Patient inclusion criteria were: age less than 35 years, body mass index (BMI) between 17 and 26 kg/m^2^, tubal and unexplained cause of infertility, first or second IVF cycle and normal partner's spermiogram according to WHO criteria.

All patients underwent controlled ovarian hyperstimulation by using short GnRH antagonist protocol. The detailed description of the IVF procedure can be found in the Supporting Information section.

### Study design

**Fig 1:**
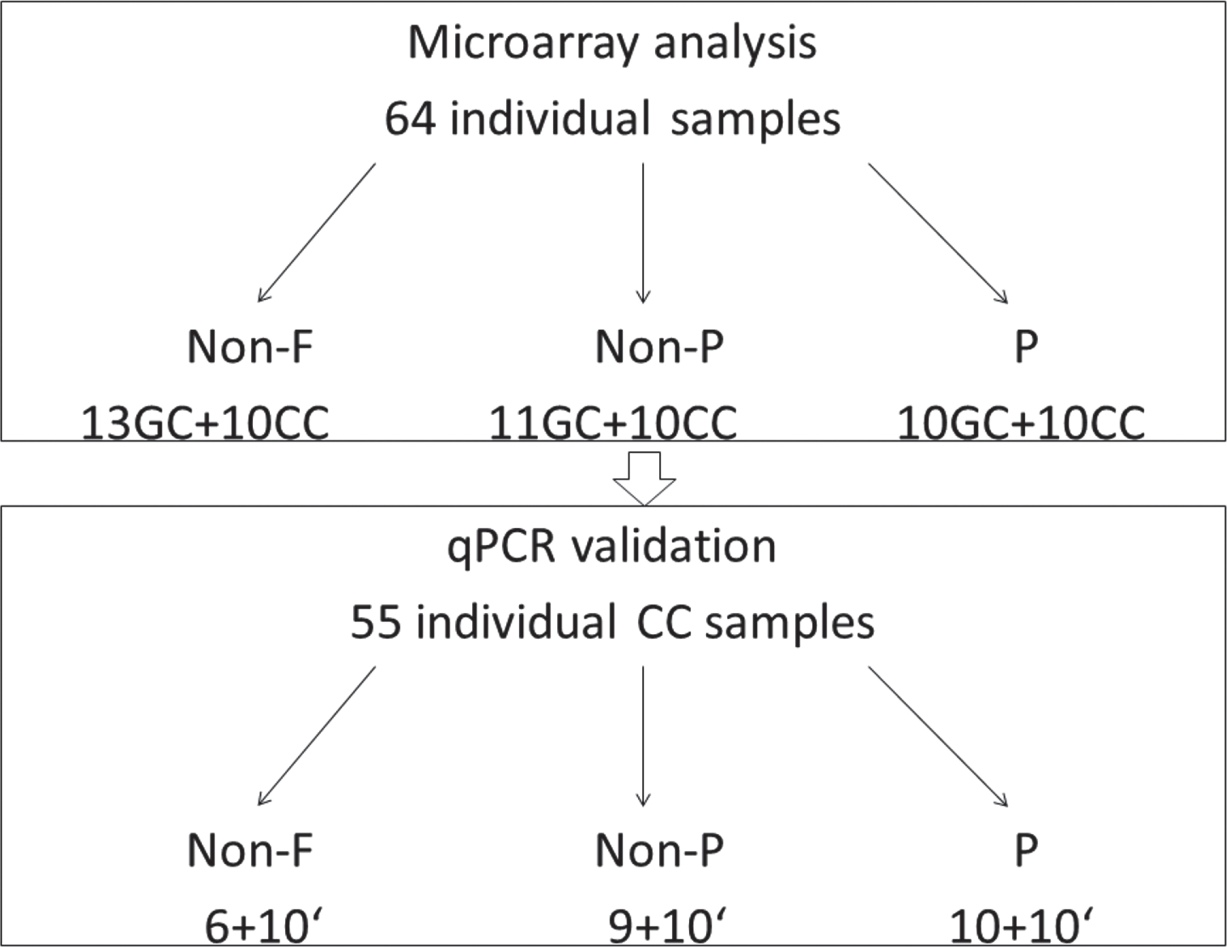
Study flowchart. Microarray analysis was performed on 64 individual cumulus and granulosa cell samples derived from 21 women; the number of the samples in each group (non-F, non-P, P) is presented. In the second stage of the study, qPCR validation was performed on 55 samples derived from 41 women; 25 samples had previously been used for microarray analysis, 30 samples were newly added to the study and derived from 20 new women. 'newly added samples; Non-F—unfertilized; non-P—fertilized, but not pregnant; P—pregnant; GC- granulosa cells; CC- cumulus cells.

We first performed genome wide gene expression analysis using microarrays on 64 individual GC and CC samples, derived from 21 women: 13 GC and 10 CC samples surrounding non-fertilized (non-F) oocytes, 11 GC and 10 CC samples surrounding fertilized oocytes, which did not lead to pregnancy after SET (non-P) and 10 GC and 10 CC samples surrounding fertilized oocytes, which led to pregnancy after SET (P). Samples of GC and CC surrounding non-F oocytes were derived from non-P and P groups; 6 GC and 5 CC samples from non-P group, 7 GC and 5 CC from P group. In the next step, quantitative real-time PCR (qPCR) validation of microarray data was performed. We have used 25 CC samples (6 non-F, 9 non-P, 10 P) that were previously included in the microarray analysis, followed by a validation on a new set of 30 CC samples (10 non-F, 10 non-P, 10 P), derived from 20 women (Fig. 1).

### Samples collection and preparation

Immediately after oocyte retrieval CC of each oocyte were removed by a needle and a glass denudation pipette (Swemed, Göteborg, Sweden), washed in phosphate-buffered saline (PBS), snap frozen in liquid nitrogen and stored at -80°C until RNA isolation.

For GC isolation Dynabeads CD45 Magnetic Beads (Invitrogen Dynal AS, Oslo, Norway) were used for depletion of CD45+ cells according to manufacturer's protocol with some adjustments. The beads were used to separate GC from leukocyte CD45+ cells to exclude the risk of contamination [12]. Follicular fluids were centrifuged at 800x g, 4°C for 10 minutes. Follicular fluids were than snap frozen in liquid nitrogen and stored at -80°C. The sediment was resuspended in 2 ml of lysis solution (0.15 M NH4Cl) to remove red blood cells, incubated for 10 minutes and centrifuged at 600x g, 4°C for 10 minutes. The supernatant was discarded and the sediment resuspended in 1 ml PBS with 0.1% bovine serum albumin (BSA) and 0.002 M ethylenediaminetetraacetic acid (EDTA). The suspension was added to the tubes containing 50 μl of Dynabeads CD45 and incubated at 4°C for 30 minutes with gentle tilting and rotation of the mixer. The tubes were pre-coated with 2 ml of Roswell Park Memorial Institute medium (RPMI) with 1% fetal calf serum (FCS) for 5 minutes. After incubation, the samples were diluted with PBS with 0.1% BSA and 0.002 M EDTA to 4 ml and placed in a magnet for 10 minutes. The supernatant was transferred to a new pre-coated tube and centifuged at 800x g, 4°C for 15 minutes. The supernatant was discarded and sediment resuspended in 1 ml of PBS. The samples were then centrifuged at 2000 x g for 5 minutes, supernatant discharged and sediment snap frozen in liquid nitrogen and stored at -80°C. The purity of the samples was checked with flow cytometry. The samples were prepared from GC suspension before isolation and compared to cell suspension after GC isolation. The contamination with leucocytes was investigated using antibodies CD45 conjugated with Fluorescin Isothiocyanate (FITC) and CD14 Phycoerythrin (PE) (both from BD Bioscience, San Jose, USA). Samples were washed and resuspended in PBS with 1% BSA and incubated with antibodies for 20 minutes. The purity of the samples was characterized by Forward Scatter (FSC) versus Side Scatter (SSC) followed by gating on CD45 and CD14. Data were collected on FACSCanto flow cytometer (BD Bioscience, San Jose, USA) and expression of various markers was assessed using FlowJo analysis software (TreeStarInc, Ashland, USA).

Before depletion of CD45+ cells there were 13% granulocytes, 10% lymphocytes and 9% monocytes present. After depletion the proportions of leukocytes were significantly lower, in average less than 3.2% of granulocytes and less than 1.5% of monocytes and lymphocytes (Table 1).

**Table 1:** Average percentages of granulosa and CD45+ cells before and after depletion.

	Granulosa cells (%)	Granulocytes (%)	Lymphocytes (%)	Monocytes (%)
**Before depletion**	66,9	14,8	9,7	9,7
**After depletion**	91,8	4,7	1,7	1,8

### RNA isolation and cDNA synthesis

RNA was extracted using TRI reagent (Sigma–Aldrich, St.Louis, USA) according to slightly modified manufacturer’s instruction. Due to small sample volume, glycogen was used as a carrier to increase RNA yield. Individual GC and CC samples were homogenized in 500 μL TRI reagent supplemented with 125 μg of glycogen (Ambion, Austin, USA). After 2 min incubation at room temperature, 100 μL chloroform was added and the sample was vortexed vigorously. RNA was precipitated from the aqueous phase with isopropanol and collected after 15 min centrifugation at 12,000x g and 4°C. RNA pellet was washed three times with 75% ethanol, dried and dissolved in 15 μL of RNAse free water. The integrity of the RNA samples was assessed with an Agilent 2100 Bioanalyzer (Agilent, Palo Alto, USA) and the total RNA quantity was measured with a Nanodrop ND-1000 spectrophotometer (Nanodrop Technologies, Wilmington, USA). cDNA was prepared from 300 ng RNA using the SuperScript Vilo reverse transcriptase (Invitrogen, Carlsbad, USA) according to manufacturer’s instructions.

### Microarray expression profiling

Microarray profiling of global gene expression on RNA samples from GC and CC was performed using Agilent SurePrint G3 Human gene expression 8x60K two-colormicroarrays (Agilent eArray design identifier: 028004), with 42.405 unique probes targeting for most of the RefSeq mRNA sequences. The experimental design comprised of co-hybridization of each tested sample with common universal reference RNA sample (Universal Human Reference RNA from Agilent). The labeling procedure was performed using Agilent’s two-color Low Input Quick Amp Labeling Kit (two-color) according to manufacturers’ instructions, labeling test samples with Cy3 and reference samples with Cy5 dye.

After hybridization, microarray slides were scanned using Agilent High Resolution Microarray Scanner System, using the manufacturers recommended scanning settings. Subsequently, microarray features were extracted using Agilent Feature Extraction (FE) software v10.7.3.1. Background was subtracted using background de-trending algorithm implemented in FE software, features with non-uniform fluorescence profiles were removed from further analyses and linear lowess intra-array normalization was performed to correct for presence of dye bias. The consistency of log ratio values was inspected using manufacturers provided spike-in probes and reproducibility was evaluated by investigating the extent of variance in the population of replicated probes on the array. The raw and normalized gene expression data were deposited to Gene Expression Omnibus (GEO) repository (http://www.ncbi.nlm.nih.gov/geo) under series accession number GSE55654.

### Statistical analyses of microarray data

All the steps described in this section, including post-processing, statistical comparisons and classification performance estimations were performed using packages from Bioconductor v2.8 project in R statistical environment version 2.13.1.

Processed fluorescence values obtained by feature extraction were imported and inspected for presence of missing values and other irregularities. Probe annotations were obtained from the Agilent’s eArray service (*earray.chem.agilent.com*). Prior to the calculation of Cy3/Cy5 log2 ratios, fluorescence values were offset by 100 units to prevent an undesirable increase in log2 ratio variance in the population of features with low fluorescence values. Quality assessment of microarray hybridizations were assessed by inspecting the signal distribution in box plots, MA plots and principal component analysis was done to identify the presence of possible batch and other confounding effects.

Statistical comparisons of expression values were evaluated using moderated t-test approach implemented in limma [21]. This test accounts for the issue of small variance contributing to undesirably large t-test values occurring for some genes, and uses Bayes inference to acknowledge similarities in gene expression profiles in subsets of genes, thereby improving prioritization of genes with differences best related to actual biological alterations. Expression values of genes were fit to a linear model, which incorporated the effect of fertilization status and tissue origin. Significance values and log2 fold-change were calculated afterwards and *p*-values were controlled for multiple testing effect by the method described by Benjamini and Hochberg [19] and implemented into the limma workflow.

### Validation of microarray data by quantitative real-time PCR

We have performed validation of microarray data results by using qPCR on both, the microarray sample set, and an independent set of samples. This was done by profiling the expression of the selected 11 genes that showed a trend toward significant transcriptional differences before the correction for multiple testing. The genes selected for validation were Calmodulin 1 (*CALM1*), Homeodomain-Interacting Protein Kinase 1 (*HIPK1*), Integral Membrane Protein 2A (*ITM2A*), Inositol-Trisphosphate 3-Kinase A (*ITPKA*), KH Domain Containing, RNA Binding, Signal Transduction Associated 3 (*KHDRBS3*), Keratin 6A (*KRT6A*), Nudix (Nucleoside Diphosphate Linked Moiety X)-Type Motif 10 (*NUDT10*), Prostaglandin-Endoperoxide Synthase 2 (*PTGS2*), T-Box 6 (*TBX6*), Transmembrane Protein 64 (*TMEM64*) and Tryptophan Rich Basic Protein (*WRB*).

Gene expression was quantified with pre-designed TaqMan Gene Expression assays (Applied Biosystems, Foster City, CA, USA). All qPCR reactions were performed in triplicates in 96-well formats in a 20 μl final volume. The measurements were performed using ABI Prism 7000 Sequence detection system (Applied Biosystems). Thermal cycling conditions were as follows: an initial step at 50°C for 2 min, denaturation at 95°C for 10 min, amplification for 40 cycles at 95°C for 15s and at 60°C for 1 min. The threshold cycle (Ct) values were then determined for each assay and were normalized to internal control glyceraldehyde-3-phosphate dehydrogenase (*GAPDH*) that was co-ran with each sample. Differences in gene expression were then calculated using the delta-deltaCt method, as previously described by Livak and Schmittgen [22]. The significance of expression differences in the two groups investigated in the validation phase was calculated using two-sample t-distribution test, differences were considered significant at α <0.05.

## Results

### Baseline characteristics

There were no significant differences in the study population and IVF cycle characteristics, except for the days of stimulation (Table 2).

**Table 2:** Patient and cycle characteristics.

Variable	Non-pregnant	Pregnant	P value
**Age (years)**	32,33±2,78	32,63±2,46	0,60
**BMI (kg/m2)**	21,17±1,73	20,56±1,14	0,40
**Days of stimulation (n)**	8,22±1,48	8,82±0,87	0,03
**Total gonadotropin dose (IU)**	1272,22±216,66	1418,18±258,14	0,75
**Oocytes retrieved (n)**	5,44±1,81	7,27±3,49	0,09
**Mature oocytes (n)**	5,00±1,87	6,55±2,84	0,26
**Immature oocytes (n)**	0,44±0,72	0,73±1,27	0,27
**Fertilization rate**	0,76±0,24	0,80±0,29	0,50

Significance of differences between the groups was assessed using independent samples t-test. Data are represented as mean±SD. SD- standard deviation.

### Gene expression profiling results

**Fig 2:**
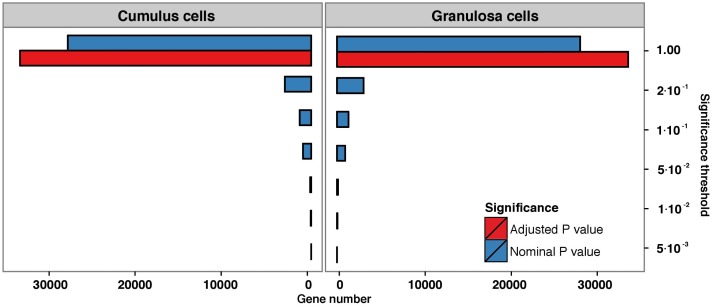
Number of genes surpassing a specified significance threshold (FDR = 0.05) in granulosa and cumulus cell lines when comparing pregnant versus non-pregnant samples. Blue bars represent nominal P value, red bars represent adjusted P value.

Comparison of transcriptional differences between non-P and P outcomes of the IVF procedure revealed that 546 genes in GC and 629 genes in CC surpassed the nominal significance threshold of 0.05. However, after the correction for multiple testing, none of the genes surpassed the adjusted significance threshold (FDR<0.05) (Fig. 2). The lists of top differentially expressed genes before the correction are presented in S1 and S2 Tables for GC and CC cells, respectively. Furthermore, the comparison of global gene expression profiles of GC and CC between non-fertilized (non-F) and fertilized (merged non-P and P) oocytes revealed, there were no differentially expressed genes after the correction for multiple testing (S1 Fig.).

**Fig 3:**
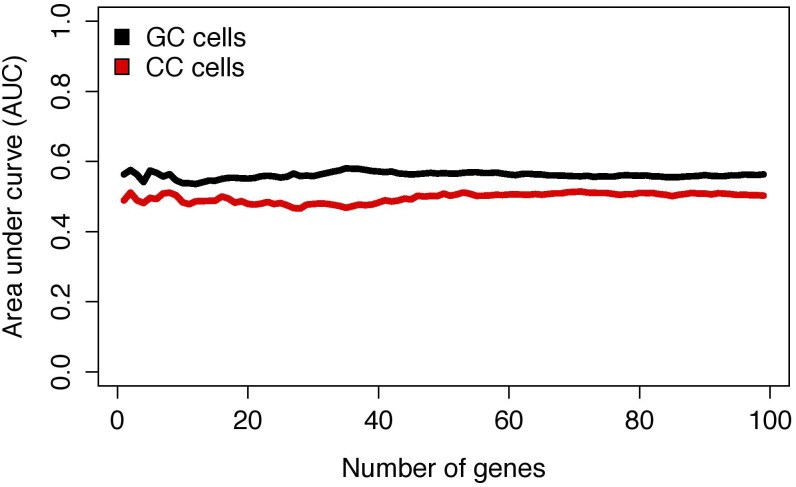
Predictive performance of expression biomarkers in granulosa and cumulus cells according to microarray data. AUC- area under the curve; GC- granulosa cells; CC- cumulus cells.

We have subsequently tested the performance of gene expression signature of GC and CC in prediction of the non-P versus P outcome. Prediction performance of gene expression based prediction model, as determined by cross-validation testing, failed to surpass AUC values of 0.5 in CC, even when the number of modeled features reached 100 genes. Similarly, in GC, AUC performance of models with up to 100 included genes failed to surpass AUC performance values of 0.6 (Fig. 3).

### qPCR validation

**Fig 4:**
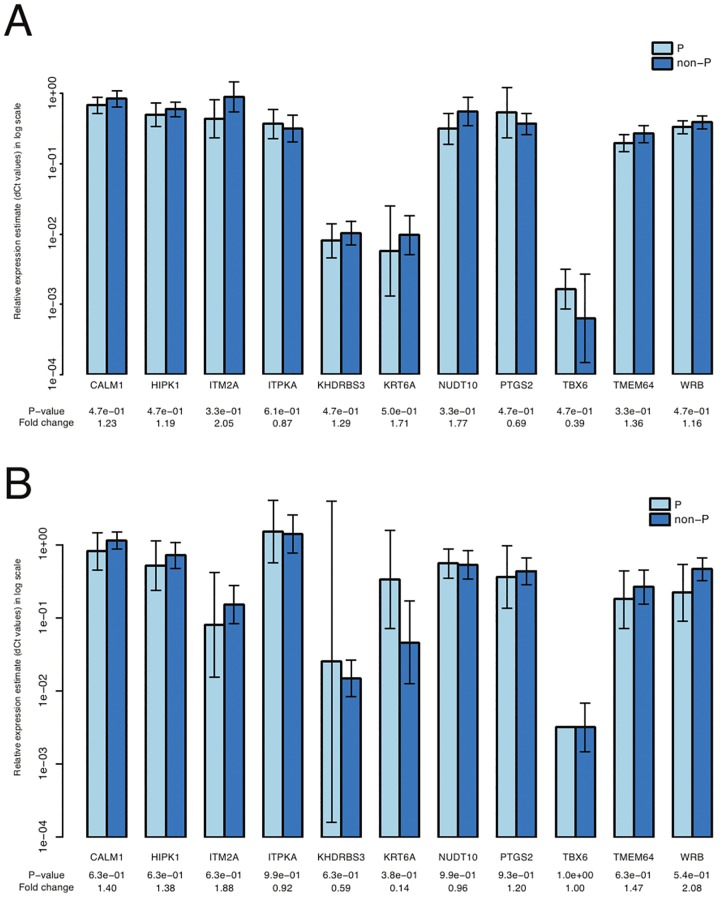
qPCR validation of microarray data. Comparison of relative mRNA expression of the genes selected for validation of microarray data between non-P and P samples. A) qPCR performed on a set of cumulus cells samples that were previously used in microarray analysis. B) qPCR performed on a novel set of cumulus cells samples. Non-P: fertilized, but not pregnant; P- pregnant.

Analysis of the expression of the twelve selected genes revealed that there were no significant differences between unfertilized and fertilized samples and non-pregnant and pregnant samples (Fig. 4 A, B). This was true for a set of samples that have previously been used for the microarray analysis and for a set of newly added samples.

## Discussion

In this study, the global gene expression profiling has shown that there are no significant gene expression differences in GC and CC surrounding fertilized and non-fertilized oocytes. Furthermore, there are no significant gene expression differences between embryos that did or did not successfully implant.

Numerous studies have tried to find a reliable method for determining oocyte and embryo viability in order to enhance the use of SET in IVF procedures by using metabolomics [23], proteomics [24,25], oocyte respiration rate [26], amino acid turnover [27] and glucose uptake [28].

With the advent of technologies for expression profiling analysis of multiple genes, several studies have tried to predict oocyte/embryo competence by analyzing genome wide GC/CC gene expression [12, 29, 30]. However, the proposed biomarkers differ between the research groups and potential biomarkers are not consistent even within the same research group. For example, in one of her most recent studies, Wathlet *et al*. [31], reported on a set of five genes (*EFNB2, GSTA4, PGR, GPX3, GSTA3*) expressed in CC to be added in a pregnancy prediction model together with patient and cycle characteristics. This model reached an accuracy of 93% for pregnancy prediction. However, in her previous study [14], there were three genes (*CAMK1D, EFNB2, STC1*) expressed in CC proposed as biomarkers of pregnancy and only *EFNB2* was retained in the pregnancy prediction model in the next study. Meanwhile, Iager *et al*. [15] performed a study in which CC samples obtained from 3 clinical sites were used for microarray and qPCR analyses. A novel set of 12 genes differentially expressed in CC was defined as predictive of pregnancy and none of them was the same as those reported by Wathlet *et al*. [14, 31]. The lack of uniformity of biomarkers probably shows that gene expression is affected by multiple variables that could be patient, treatment, etiology and laboratory specific and this could be the reason why the differences in gene expression cannot be replicable.

The comparison of gene expression of GC and CC between embryos that did or did not implant, revealed there were 546 (GC) and 629 (CC) differentially expressed genes. However, after the correction for multiple testing none of the genes surpassed the adjusted significance threshold in either group of the cell types. The control of false positive rates is essential in microarray experiments as it is well known that due to the large number of genes on the microarray and the large number of tests performed, a considerable number of genes may show differential expression simply by chance [32, 33]. Without regard of this effect, the large numbers of statistical hypotheses tested on the same study sample would significantly increase the representation of random false-positive calls among the reported significant results, resulting in poor replication rate between different microarray studies. Controlling for the false positive rate, as outlined by Benjamini and Hoechberg [19], offers a method to control such situations by estimating the probability of false positive results at varying nominal significance levels and thus provides information on the number of false-positive results among the genes with reported significant alterations.

For validation of microarray data by qPCR, a set of genes that showed a trend toward being significantly differentially expressed before the correction for multiple testing, was selected. Results of validation were in consent with microarray data and none of the genes surpassed the significance threshold. This was true for the set of samples that had previously been used for microarray analysis and a novel set of samples.

The results of our study differ from the results of other studies, which proposed different genes as biomarkers of pregnancy [12, 13, 14, 15, 34, 35]. The reason why we did not find differentially expressed genes could derive from the fact that we have tried to exclude factors that could affect gene expression in GC and CC and thus present biased results in biomarker search by strict inclusion criteria and study design. Only younger patients, under the age of 35, were included in the study, as it is known that gene expression is affected by patients age [16]. In addition, the possibility of different stimulation therapy being the reason for gene expression differences [17] was excluded by the use of only one stimulation protocol. With the inclusion of patients with tubal or unexplained cause of infertility, we excluded the possibility of gene expression differences deriving from primary disease such as PCO [36] or endometriosis [37, 38]. By analyzing individual GC and CC samples, we have gained detailed information about gene expression for each individual follicle, as it is known that pooling of samples can mask the detailed intrafollicular conditions [39]. Additionaly, by elective single embryo transfer we precisely knew which embryo did and which did not implant. These assumptions are further supported by the finding that the principal source of biases in microarray experiments derives from individual differences between samples and experimental design and not from the measuring technique [40]. With a limited number of microarray experiments performed, even small differences that arise from an individual sample, the experimental condition or any other study design parameter and not from the end-point studied, may lead to a false positive result. Such false positives however, can not be controlled by any statistical method.

Furthermore, the comparison of gene expression profile of GC and CC between unfertilized and fertilized oocytes showed no significant differences. This finding is in agreement with that of Devjak *et al*. where there were no differentially expressed genes between CC surrounding mature unfertilized oocytes and oocytes that developed to blastocysts [41].

It is well known that embryo implantation in the uterus depends on various factors such as: embryo's chromosomal status [42], endometrial receptivity [43], embryo culture conditions [44], embryo transfer technique [45] and patients' lifestyle and pre-existing conditions [46]. These variables affect embryo implantation on the level that is not related to gene expression in CC and GC and could be one of the reasons for the lack of uniformity of biomarkers proposed by different groups. Keeping this in mind, perhaps we will never be able to understand this complex process by focusing only on gene expression in somatic cells of ovarian follicles. It is likely, that embryo implantation is a result of the presenece of euploid oocytes/embryos and a balanced expression of many genes that are expressed not only in GC/CC but also in endometrium and this concept should be kept in mind for the future studies.

In conclusion, on our set of GC and CC samples derived from a highly homogenous group of patients we did not find gene expression signatures specific for successful embryo implantation and oocyte fertilization after rigorous statistical evaluation of genome-wide gene expression analysis.

## Supporting Information

S1 Fig Number of genes surpassing specified significance threshold (FDR = 0.05) in granulosa and cumulus cell lines when comparing non-fertilized (non-F) versus fertilized (non-P+P) samples. Blue bars represent nominal P value, red bars represent adjusted P value.
(TIF)

S1 File (DOCX)

S1 Table Top differentially expressed genes in GC between non-pregnant versus pregnant samples. (DOCX)

S2 Table Top differentially expressed genes in CC between non-pregnant versus pregnant samples before the correction for multiple testing. (DOCX)
