# Supplementary figures and images for: No Specific Gene Expression Signature in Human Granulosa and Cumulus Cells for Prediction of Oocyte Fertilisation and Embryo Implantation
Source: PLoS One. 2015 Mar 13;10(3):e0115865. doi: 10.1371/journal.pone.0115865 (PMC4359149; doi:10.1371/journal.pone.0115865)

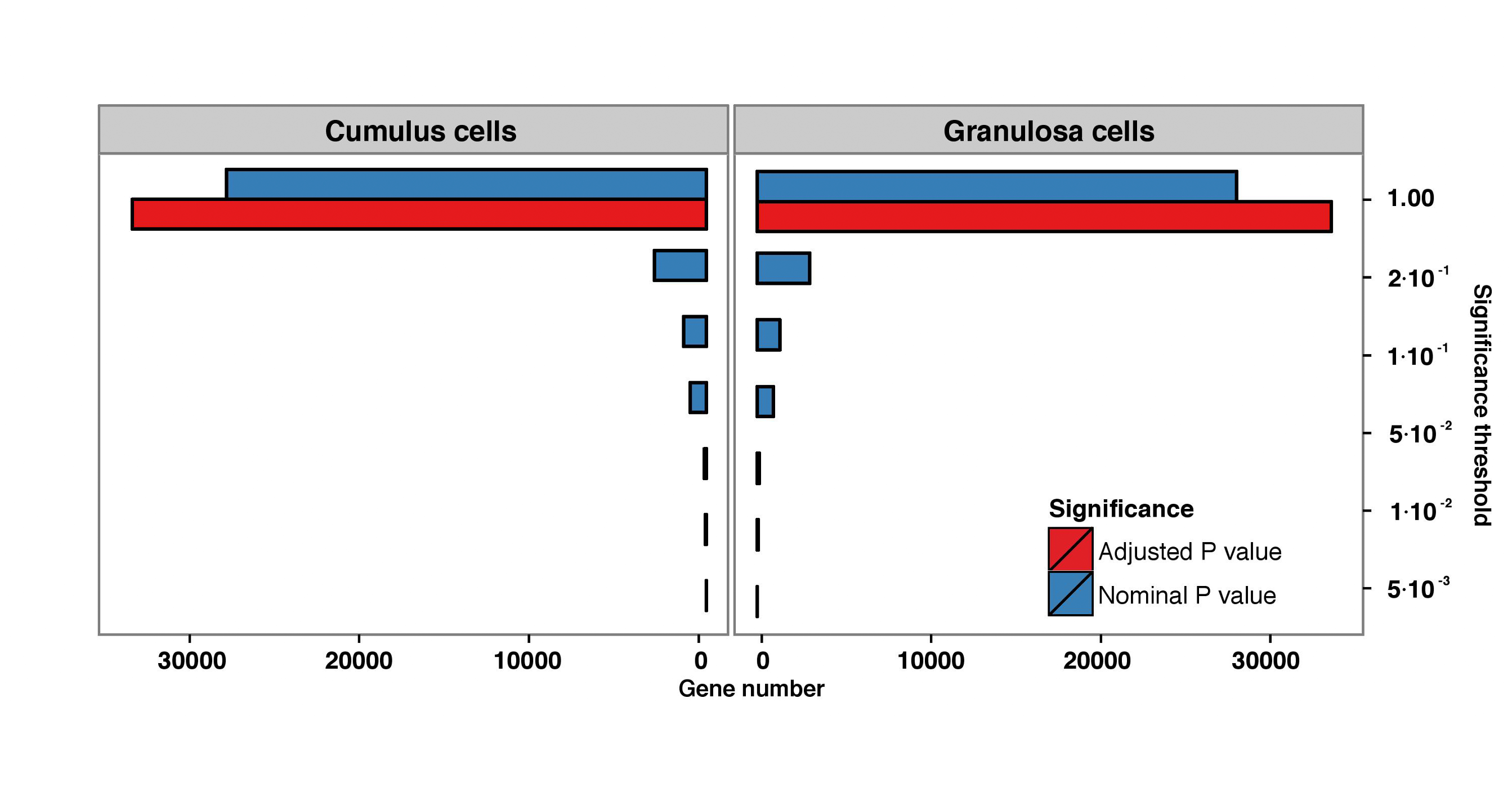

Supplement: S1 Fig — Blue bars represent nominal P value, red bars represent adjusted P value. (TIF) [file pone.0115865.s001.tif]
