# Supplementary material for: No Specific Gene Expression Signature in Human Granulosa and Cumulus Cells for Prediction of Oocyte Fertilisation and Embryo Implantation
Source: PLoS One. 2015 Mar 13;10(3):e0115865. doi: 10.1371/journal.pone.0115865 (PMC4359149; doi:10.1371/journal.pone.0115865)
Supplement: S1 File — (DOCX) [file pone.0115865.s002.docx]

**IVF procedure**

All patients underwent controlled ovarian hyperstimulation by administration of recombinant FSH (Puregon; Schering Plough, New Jersey, USA) with GnRH antagonist cetrorelix acetate (Cetrotide; Asta Medica AG, Frankfurt, Germany). Vaginal ultrasound examination was used to monitor follicular development. Final follicular maturation was induced by administering 10,000 IU of human chorionic gonadotrophin (hCG) (Pregnyl; N.V. Organon, Oss, the Netherlands) when at least three follicles were ≥17 mm. Ultrasound guided transvaginal retrieval of follicles was performed 34–36 h later. Each follicle was punctured and follicular fluid aspirated separately. After the ovarian puncture, cumulus-oocyte complexes (COC) were removed from follicular fluid. Cumulus cells of each oocyte were immediately removed. Oocytes were then fertilized (classical IVF) and cultured individually, allowing a correlation with their respective GC and CC. After 18-20 hours, oocyte fertilization status was assessed. Fertilized oocytes expressed two pronuclei and two polar bodies, whereas unfertilized oocytes did not. Fertilized oocytes were individually cultured for 5 days in the Universal IVF Medium followed by BlastAssist System (M1 and M2, Origio, Denmark). Elective single embryo transfer (SET) of the blastocyst was performed on day 5 of IVF procedure. Supernumerary blastocsyts were cryopreserved. Biochemical pregnancy was determined by measuring of βhCG 14 days after SET, clinical pregnancy was determined as the presence of gestational sac and fetal heartbeat by ultrasound examination 6 weeks after SET.
