# Supplementary material for: No Specific Gene Expression Signature in Human Granulosa and Cumulus Cells for Prediction of Oocyte Fertilisation and Embryo Implantation
Source: PLoS One. 2015 Mar 13;10(3):e0115865. doi: 10.1371/journal.pone.0115865 (PMC4359149; doi:10.1371/journal.pone.0115865)
Supplement: S1 Table — (DOCX) [file pone.0115865.s003.docx]

| **GeneName** | **Description** | **logFC** | **P.Value** |
| --- | --- | --- | --- |
| lincRNA:chr11:123940940-123956165_F | lincRNA:chr11:123940940-123956165 forward strand | -0,2 | 2,5E-04 |
| lincRNA:chr8:2522118-2527693_R | lincRNA:chr8:2522118-2527693 reverse strand | -0,2 | 3,1E-04 |
| THC2642375 | tc\|XM_363028 predicted protein {Magnaporthe grisea 70-15} (exp=0; wgp=1; cg=0), partial (4%) | -0,2 | 3,3E-04 |
| lincRNA:chr6:106810232-106852232_F | lincRNA:chr6:106810232-106852232 forward strand | -0,4 | 4,1E-04 |
| lincRNA:chr8:130231743-130741043_R | lincRNA:chr8:130231743-130741043 reverse strand | -0,4 | 4,4E-04 |
| RSPO4 | R-spondin 4 | -0,2 | 5,6E-04 |
| ELMOD1 | ELMO/CED-12 domain containing 1 | -0,2 | 5,8E-04 |
| TNFRSF10A | Tumor necrosis factor receptor superfamily, member 10a | -0,3 | 6,5E-04 |
| ESM1 | Endothelial cell-specific molecule 1 | -0,2 | 6,7E-04 |
| NNMT | Nicotinamide N-methyltransferase | 0,7 | 7,5E-04 |
| LOC644192 | PREDICTED: Homo sapiens hypothetical LOC644192 (LOC644192), miscRNA | -0,3 | 8,3E-04 |
| lincRNA:chr19:56791970-56826162_F | lincRNA:chr19:56791970-56826162 forward strand | -0,3 | 8,3E-04 |
| LOC648691 | Homo sapiens uncharacterized LOC648691 (LOC648691), non-coding RNA | -0,3 | 8,6E-04 |
| VAX1 | Ventral anterior homeobox 1 | -0,3 | 8,7E-04 |
| C8orf48 | Chromosome 8 open reading frame 48 | -0,2 | 8,8E-04 |
| lincRNA:chr3:185544131-185550706_R | lincRNA:chr3:185544131-185550706 reverse strand | -0,3 | 9,8E-04 |
| GHRH | Growth hormone releasing hormone | -0,2 | 1,2E-03 |
| GRM2 | Glutamate receptor, metabotropic 2 | -0,2 | 1,3E-03 |
| ENST00000409554 | ens\|serine peptidase inhibitor-like, with Kunitz and WAP domains 1 (eppin) [Source:HGNC Symbol;Acc:15932] | -0,7 | 1,3E-03 |
| lincRNA:chr12:5231714-5344139_F | lincRNA:chr12:5231714-5344139 forward strand | -0,3 | 1,3E-03 |
| CSPG5 | Chondroitin sulfate proteoglycan 5 (neuroglycan C) | -0,2 | 1,4E-03 |
| lincRNA:chr4:13067152-13347902_R | lincRNA:chr4:13067152-13347902 reverse strand | -0,8 | 1,5E-03 |
| AY338954 | Prostate-specific P775P mRNA sequence | -0,3 | 1,5E-03 |
| LTBP2 | Latent transforming growth factor beta binding protein 2 | -0,2 | 1,5E-03 |
| WSCD1 | WSC domain containing 1 | -0,3 | 1,8E-03 |
| ZC3H8 | Zinc finger CCCH-type containing 8 | -0,3 | 1,8E-03 |
| SLC11A2 | Solute carrier family 11 (proton-coupled divalent metal ion transporters), member 2 | -0,4 | 1,8E-03 |
| ENST00000507496 | tc\|2AFD_A Chain A, Solution Structure Of Asl1650, An Acyl Carrier Protein From Anabaena Sp. Pcc 7120 With A Variant Phosphopantetheinylation-Site Sequence. {Nostoc sp. PCC 7120} (exp=-1; wgp=0; cg=0), partial (19%) | -0,2 | 1,9E-03 |
| GPLD1 | Glycosylphosphatidylinositol specific phospholipase D1 | -0,1 | 2,0E-03 |
| lincRNA:chrX:10933204-10941554_R | lincRNA:chrX:10933204-10941554 reverse strand | -0,2 | 2,2E-03 |
| BAAT | Homo sapiens bile acid CoA: amino acid N-acyltransferase (glycine N-choloyltransferase) (BAAT), transcript variant 1, mRNA | -0,2 | 2,2E-03 |
| LOC283788 | Homo sapiens FSHD region gene 1 pseudogene (LOC283788), non-coding RNA | -0,3 | 2,3E-03 |
| PROM2 | Homo sapiens prominin 2 (PROM2), transcript variant 3, mRNA | -1,0 | 2,4E-03 |
| lincRNA:chr7:32955831-32961444_R | lincRNA:chr7:32955831-32961444 reverse strand | -0,3 | 2,6E-03 |
| lincRNA:chr18:72865737-72876737_R | lincRNA:chr18:72865737-72876737 reverse strand | -0,3 | 2,6E-03 |
| GRB10 | Homo sapiens growth factor receptor-bound protein 10 (GRB10), transcript variant 4, mRNA | 0,8 | 2,7E-03 |
| MRPL23-AS1 | Homo sapiens MRPL23 antisense RNA 1 (non-protein coding) (MRPL23-AS1), non-coding RNA | -0,2 | 2,8E-03 |
| lincRNA:chr17:15385375-15392225_R | lincRNA:chr17:15385375-15392225 reverse strand | 0,3 | 2,9E-03 |
| lincRNA:chr16:72281724-72432024_R | lincRNA:chr16:72281724-72432024 reverse strand | -0,5 | 2,9E-03 |
| lincRNA:chr1:177966002-178004377_F | lincRNA:chr1:177966002-178004377 forward strand | -0,4 | 2,9E-03 |
| GATAD2B | Homo sapiens GATA zinc finger domain containing 2B (GATAD2B), mRNA | 0,5 | 3,0E-03 |
| lincRNA:chr19:56789963-56824563_R | lincRNA:chr19:56789963-56824563 reverse strand | -0,3 | 3,1E-03 |
| lincRNA:chr12:130559722-130642097_F | lincRNA:chr12:130559722-130642097 forward strand | -0,3 | 3,1E-03 |
