# Supplementary material for: No Specific Gene Expression Signature in Human Granulosa and Cumulus Cells for Prediction of Oocyte Fertilisation and Embryo Implantation
Source: PLoS One. 2015 Mar 13;10(3):e0115865. doi: 10.1371/journal.pone.0115865 (PMC4359149; doi:10.1371/journal.pone.0115865)
Supplement: S2 Table — (DOCX) [file pone.0115865.s004.docx]

| **GeneName** | **Description** | **logFC** | **P.Value** |
| --- | --- | --- | --- |
| lincRNA | lincRNA:chr7:105525814-105538089 forward strand | -0,4 | 5,2E-05 |
| lincRNA | lincRNA:chr15:58549646-58623663 forward strand | 0,3 | 9,8E-05 |
| ANKRD33B | Ankyrin repeat domain 33B | -0,2 | 4,5E-04 |
| MTA1 | Metastasis associated 1 | -0,3 | 4,8E-04 |
| NUDT10 | Nudix (nucleoside diphosphate linked moiety X)-type motif 10 | 1,1 | 5,9E-04 |
| KRT6A | Keratin 6A | -2,8 | 8,0E-04 |
| KAT2A | K(lysine) acetyltransferase 2A | -0,5 | 8,9E-04 |
| lincRNA | lincRNA:chr10:29699919-29718494 reverse strand | -0,4 | 9,3E-04 |
| TBX6 | T-box 6 | -0,3 | 9,8E-04 |
| HIPK1 | Homeodomain interacting protein kinase 1 | 0,2 | 1,1E-03 |
| RPS15 | Ribosomal protein S15 | -0,2 | 1,5E-03 |
| C9orf64 | Chromosome 9 open reading frame 64 | 0,2 | 1,9E-03 |
| RANBP1 | RAN binding protein 1 | -0,2 | 1,9E-03 |
| FCRL6 | Fc receptor-like 6 | 0,3 | 1,9E-03 |
| PCDH18 | Protocadherin 18 | 0,2 | 2,1E-03 |
| ALOX15 | Arachidonate 15-lipoxygenase | 0,2 | 2,2E-03 |
| STK38 | Serine/threonine kinase 38 | 0,4 | 2,2E-03 |
| ARL6IP1 | ADP-ribosylation factor-like 6 interacting protein 1 | 0,4 | 2,2E-03 |
| lincRNA | lincRNA:chr1:229378602-229388877 forward strand | -0,2 | 2,4E-03 |
| LYPD2 | LY6/PLAUR domain containing 2 | 0,2 | 2,6E-03 |
| KRT13 | Keratin 13 | -2,1 | 2,6E-03 |
| MAT2B | Methionine adenosyltransferase II, beta | 0,3 | 2,7E-03 |
| WRB | Tryptophan rich basic protein | 0,2 | 2,9E-03 |
| PSPH | Phosphoserine phosphatase | 1,6 | 2,9E-03 |
| NDUFA3 | NADH dehydrogenase (ubiquinone) 1 alpha subcomplex, 3, 9kDa | -0,2 | 2,9E-03 |
| KANSL3 | KIAA1310 | 0,3 | 3,0E-03 |
| CYP4X1 | Cytochrome P450, family 4, subfamily X, polypeptide 1 | 0,2 | 3,0E-03 |
| SPRED2 | Sprouty-related, EVH1 domain containing 2 | 0,3 | 3,0E-03 |
| MAP2K6 | Mitogen-activated protein kinase 6 | 0,4 | 3,2E-03 |
| lincRNA | lincRNA:chr4:2463252-2468502 forward strand | 0,3 | 3,3E-03 |
| lincRNA | lincRNA:chr18:14053400-14102850 forward strand | 0,3 | 3,3E-03 |
| CALM1 | Calmodulin 1 (phosphorylase kinase, delta) | 0,5 | 3,3E-03 |
| MSH5 | MutS homolog 5 (E. coli) | -0,2 | 3,6E-03 |
| NRIP3 | Nuclear receptor interacting protein 3 | 0,5 | 3,6E-03 |
| SNRPA | Small nuclear ribonucleoprotein polypeptide A | -0,2 | 3,8E-03 |
| DDX56 | DEAD (Asp-Glu-Ala-Asp) box polypeptide 56 | -0,2 | 3,9E-03 |
| lincRNA:chr13:27831250-27836500_R | lincRNA:chr13:27831250-27836500 reverse strand | 0,2 | 3,9E-03 |
| PYGM | Phosphorylase, glycogen, muscle | 0,3 | 4,0E-03 |
| CWC25 | CWC25 spliceosome-associated protein homolog (S. cerevisiae) | 0,2 | 4,0E-03 |
| SLC16A4 | Solute carrier family 16, member 4 (monocarboxylic acid transporter 5) | 0,5 | 4,0E-03 |
| GAFA2 | FGF-2 activity-associated protein 2 | 0,3 | 4,0E-03 |
| C19orf6 | Chromosome 19 open reading frame 6 | -0,2 | 4,0E-03 |
| lincRNA | lincRNA:chr10:17250419-17261819 reverse strand | -0,2 | 4,0E-03 |
| CDNF | Cerebral dopamine neurotrophic factor | 0,3 | 4,0E-03 |
| lincRNA | lincRNA:chr5:131806278-131808704 forward strand | -0,3 | 4,0E-03 |
